# S253. PERSON-CENTERED PSYCHOSIS CARE (PCPC) IN AN INPATIENT SETTING: PATIENT OUTCOMES

**DOI:** 10.1093/schbul/sby018.1040

**Published:** 2018-04-01

**Authors:** Anneli Goulding, Katarina Allerby, Lilas Ali, Margda Waern

**Affiliations:** University of Gothenburg

## Abstract

**Background:**

The person-centered care approach has been little tested in inpatient settings for persons with schizophrenia and similar psychoses. We developed a staff educational intervention, Person-Centered Psychosis Care (PCPC) tailored to our care setting (4 hospital wards for persons with psychoses, 43 beds). The intervention was co-created by professionals, patients, and researchers using a participatory approach. There was a focus on the patient’s narrative, the creation of partnership between staff and patient, an agreement between staff and patient concerning care, and a bridging of inpatient and outpatient care and support. The present study aims to describe patient outcomes associated with PCPC.

**Methods:**

The study had a before and after design. Before the PCPC intervention started, questionnaire data was collected from 50 inpatients shortly before discharge. Post intervention data are currently under collection (anticipated n=50). The primary outcome measure is self-reported empowerment (Empowerment Scale, Range 0–112) and the secondary measure is consumer satisfaction (UKU-ConSat Rating Scale, converted to range between 11 and 77). Participants also complete questionnaires related to possible confounding variables such as overall health (EQ-5D), symptom burden (PANSS), and functional ability (GAF).

**Results:**

The participants (46% women) included in the pre-intervention sample had a mean age of 47.5 years (SD=14.5). The total mean empowerment score for the pre-intervention sample was 82.6 (SD=8.1) whereas the mean consumer satisfaction score was 51.5 (SD=12.9). There were no statistically significant gender differences regarding empowerment or consumer satisfaction. There were no significant correlations between age, any of the confounding variables, and empowerment and consumer satisfaction. We will present results from comparisons between the pre- and post-intervention groups regarding empowerment and consumer satisfaction.

**Discussion:**

The before and after design has its limitations, but if the PCPC intervention proves beneficial, such a model could be tested with a cluster randomized study design.

